# Tracking the impact of bottom trawling on benthic habitat status

**DOI:** 10.1111/cobi.70303

**Published:** 2026-04-22

**Authors:** Maider Plaza‐Morlote, Ana García‐Alegre, Alberto Serrano, Ulla Fernández‐Arcaya, Antonio Punzón, Ana Rodríguez‐Rúa, Sara Roman, Ana Magro, Marina Delgado, Juan Gil, José Manuel González‐Irusta

**Affiliations:** ^1^ Instituto Español de Oceanografía (COST IEO‐CSIC) Centro Oceanográfico de Santander Santander Spain; ^2^ Universidad de Vigo, Campus Do Mar Facultad de Ciencias del Mar Vigo Spain; ^3^ Instituto Español de Oceanografía (CNIEO‐CSIC) Centro Oceanográfico de Cádiz Cádiz Spain

**Keywords:** benthic habitats, bottom trawling, GES, good environmental status, Marine Strategy Framework Directive, MSFD, quality thresholds, sentinels of the seabed indicator, buen estado ambiental, Directiva Marco sobre la Estrategia Marina, arrastre de fondo, GES, hábitats bentónicos, indicador «Sentinels of Seabed», MSFD, pesca de arrastre de fondo, umbrales de calidad

## Abstract

Bottom trawling is the most widespread physical disturbance to marine benthic habitats, yet broadscale assessments of its impact remain limited. We developed a quantitative framework to evaluate trawling effects on benthic habitats at broad spatial scales based on the sentinels of seabed (SoS) indicator. We applied this approach across five broad benthic habitat types in the Bay of Biscay and the Gulf of Cádiz, two regions that are subject to substantial trawling pressure. Using data from over 2000 monitoring locations, we assessed habitat sensitivity to trawling through generalized additive models (GAMs) and the SoS indicator, which quantifies benthic habitat quality based on the proportion of sentinel species (i.e., species typical of the habitat and sensitive to the pressure). We also sought to establish scientifically grounded quality thresholds to assess bottom‐trawling impacts by applying three complementary approaches: distance to degradation, natural variation, and detectable change. We conducted a temporal analysis to evaluate changes in trawling impacts over time. Impact assessments varied depending on the method used to determine thresholds, although the natural variation and distance to degradation approaches yielded consistent results. Despite the methodological differences, all approaches identified offshore circalittoral mud and offshore circalittoral mixed sediments as the most affected habitats. The Gulf of Cádiz had more overall impacts than northern Spain. Impacts decreased significantly across all benthic habitats in association with declining trawling intensity, although extensive areas remained at risk. Our approach provides a scalable and robust way to assess seabed condition at broad spatial scales. By establishing critical benchmarks for habitat quality and pressure, it offers a scientifically sound tool to inform national and regional marine management strategies. The approach supports the implementation of environmental policies, such as the European Union Marine Strategy Framework Directive, and contributes to achieving good environmental status.

## Abstract (es)

Seguimiento al impacto de la pesca de arrastre de fondo sobre el estado de los hábitats bentónicos

**Resumen:**

La pesca de arrastre de fondo es la perturbación física más extendida de los hábitats bentónicos marinos; sin embargo, las evaluaciones a gran escala de su impacto siguen siendo limitadas. Desarrollamos un marco cuantitativo para evaluar los efectos de la pesca de arrastre sobre los hábitats bentónicos a gran escala espacial con base en el indicador «Sentinels of Seabed» (SoS). Aplicamos este enfoque a cinco grandes tipos de hábitats bentónicos en el golfo de Vizcaya y el golfo de Cádiz, dos regiones sometidas a una presión considerable por la pesca de arrastre. Utilizamos datos de más de 2,000 puntos de seguimiento, evaluamos la sensibilidad del hábitat a la pesca de arrastre mediante modelos aditivos generalizados (MAG) y el indicador SoS, que cuantifica la calidad del hábitat bentónico con base en la proporción de especies centinela (es decir, especies típicas del hábitat y sensibles a la presión). También tratamos de establecer umbrales de calidad con base científica para evaluar los impactos de la pesca de arrastre de fondo mediante la aplicación de tres enfoques complementarios: distancia a la degradación, variación natural y cambio detectable. Realizamos un análisis temporal para evaluar los cambios en los impactos de la pesca de arrastre a lo largo del tiempo. Las evaluaciones de impacto variaron en función del método utilizado para determinar los umbrales, aunque los enfoques basados en la variación natural y en la distancia a la degradación arrojaron resultados coherentes. A pesar de las diferencias metodológicas, todos los enfoques identificaron los lodos circalitorales de alta mar y los sedimentos mixtos circalitorales de alta mar como los hábitats más afectados. El golfo de Cádiz presentó más impactos en general que el norte de España. Los impactos disminuyeron significativamente en todos los hábitats bentónicos a medida que disminuía la intensidad de la pesca de arrastre, aunque amplias zonas seguían estando en riesgo. Nuestro enfoque ofrece una forma escalable y sólida para evaluar el estado del lecho marino a gran escala espacial. Al establecer puntos de referencia críticos para la calidad del hábitat y la presión, ofrece una herramienta científicamente sólida para fundamentar las estrategias de gestión marina nacionales y regionales. El enfoque respalda la aplicación de políticas medioambientales, como la Directiva Marco sobre la Estrategia Marina de la Unión Europea, y contribuye a alcanzar un buen estado medioambiental.

## INTRODUCTION

The functioning and integrity of marine ecosystems are essential for human health and well‐being (European Marine Board, 2013; Fleming et al., 2023; Gollan et al., 2019; Whitmee et al., 2015). Despite this recognition, human activities at sea continue to intensify, increasing pressures on ocean health and compromising the provision of essential marine ecosystem services (Constanza et al., 2014; Culhane et al., 2019; Halpern et al., 2008). To counteract these impacts, national and international policies have been established to balance ecosystem protection with sustainable use. In Europe, several directives have been introduced to safeguard marine ecosystems, including the Habitat Directive (92/43/EEC), the Water Framework Directive (2000/60/CE), and the Maritime Spatial Planning Directive (2014/89/EU). Among these, the Marine Strategy Framework Directive (MSFD) (Directive 2008/56/EC) is particularly comprehensive and ambitious, as is the Nature Restoration Law (2024/1991/EU). The primary objective of the MSFD is to regulate human activities and mitigate their pressures to ensure the long‐term sustainable use of marine resources (Bigagli, 2015; Borja et al., 2010). The MSFD defines “good environmental status” (GES) through 11 qualitative descriptors, with Descriptor 1 (biodiversity) and Descriptor 6 (seafloor integrity) being particularly relevant to seabed habitats. However, a major challenge in assessing benthic habitats under this framework lies in the absence of scientifically justified ecological quality thresholds, making it difficult to determine habitat condition and hampering effective implementation.

Among the various pressures affecting benthic ecosystems (Clark et al., 2016; Harris, 2020), mobile bottom‐contact fishing is the primary driver of physical disturbance in European Atlantic seabeds due to its widespread and intense distribution down to 800 m water depth (Eigaard et al., 2017; Foden et al., 2011; Smith et al., 2023). The Bay of Biscay and the Gulf of Cadiz are no exception, with trawling pressure affecting more than 90% of sedimentary habitats at a grid resolution of 0.05° × 0.05° (Fernandez‐Arcaya et al., 2024).

Assessing the environmental status of benthic habitats requires indicators sensitive to physical pressures (ICES, 2022b; van Denderen et al., 2024) and well‐defined ecological quality thresholds to determine when a habitat transitions from a good to a degraded state (Grumbine, 1994; Hiddink et al., 2023; ICES, 2022a, 2022b). Although progress has been made in developing benthic indicators to assess trawling impacts (van Denderen et al., 2024), many assessments still rely on arbitrary thresholds without strong ecological justification (ICES, 2022a, 2022b; Matear et al., 2023; Serrano et al., 2022; Smith et al., 2023). Without robust thresholds, these indicators remain largely descriptive, providing snapshots of variability rather than actionable insights for management decisions.

To address this gap, we applied, for the first time, a structured approach to setting ecological thresholds based on a well‐established and pressure‐sensitive indicator: sentinels of seabed (SoS) (Serrano et al., 2022). This indicator is also referred to as BH1 in the Oslo–Paris Convention (OSPAR) (Plaza‐Morlote et al., 2023). We selected SoS for its robust quantitative methodology (Serrano et al., 2022), its demonstrated sensitivity to trawling pressures across multiple regions (ICES, 2022b; Plaza‐Morlote et al., 2023; van Denderen et al., 2024), and its recognition as an OSPAR common indicator. To establish scientifically grounded thresholds, we tested three widely recognized methodological approaches: distance to degradation (ICES, 2022a; Plaza‐Morlote et al., 2023), natural variation (Hiddink et al., 2023; ICES, 2022a; Landres et al., 1999; Rossberg et al., 2017), and detectable change (Hiddink et al., 2023; ICES, 2022a, 2022b). Each approach provides a different perspective on defining ecological thresholds, which contributes to a more comprehensive assessment of trawling‐induced adverse effects.

The Bay of Biscay and the Gulf of Cadiz offer an ideal case study for testing this assessment framework. These regions benefit from long‐standing and extensive monitoring programs that offer valuable information on invertebrate abundance across a pressure gradient. This makes them particularly suitable for robust empirical analysis of the relationship between trawling intensity and benthic status. Although previous studies have explored the spatiotemporal distribution of trawling pressure in these habitats (Fernandez‐Arcaya et al., 2024), assessments of seabed were limited to specific areas or habitat types (ICES, 2022b; Serrano et al., 2022; van Denderen et al., 2024), often relying on arbitrary thresholds (Serrano et al., 2022).

By integrating a pressure‐sensitive and scientifically validated indicator with multiple threshold‐setting methodologies, we sought to provide a robust framework for assessing trawling impacts on benthic habitats. Rather than aiming to develop an idealized or purely academic model, the goal was to deliver a practical and ecologically meaningful tool that addresses real‐world management needs while accounting for the environmental variability inherent to the assessment units. Specifically, we aimed to determine the acceptable level of state change in benthic habitats based on scientifically grounded quality thresholds; propose a standardized method for national and regional benthic habitat assessments; and establish ecological benchmarks for bottom‐trawling impacts on benthic habitats in Spanish Atlantic waters. This study provides the first threshold‐based assessment of trawling impacts on benthic habitats in Spanish waters, offering a scalable and evidence‐based framework to support ecosystem‐based management and inform policy decisions under the MSFD.

## METHODS

We estimated the extent of adverse effects on benthic habitats caused by bottom trawling (Appendix S1). Mobile bottom fishing pressure was quantified for each year and grid cell based on the swept area ratio (SAR) (ICES, 2019). The distribution of MSFD broad habitat types (BHTs) (EMODnet, 2021) (defined below) was then used as the spatial framework for integrating habitat information with bottom‐trawling pressure. The BHT polygons were first used to delineate and mask the SAR layer, which produced a raster output in which fishing pressure was spatially constrained by habitat boundaries. To preserve the original habitat extent in the final output, the SAR layer was upscaled from 0.05° × 0.05° to 0.01° × 0.01° with a nearest‐neighbor algorithm in which SAR values in each grid cell were held constant. Benthic sampling stations were overlaid on the SAR grid and the BHT polygons to assign each observation a corresponding BHT and SAR value for the grid cell in which it occurred. These habitat and pressure attributes were then combined within the SoS indicator to quantify the proportion of sentinel species, which reflected benthic environmental status. Finally, three threshold‐setting methods were applied to evaluate the adverse effects of trawling on BHTs. These analyses covered the period from 2016 to 2022, allowing an assessment of progress since the second EU MSFD Article 8 report in 2018 and a comparison with the previous 6‐year period (2010–2015).

### Study sites and seabed habitat component

**FIGURE 1:**
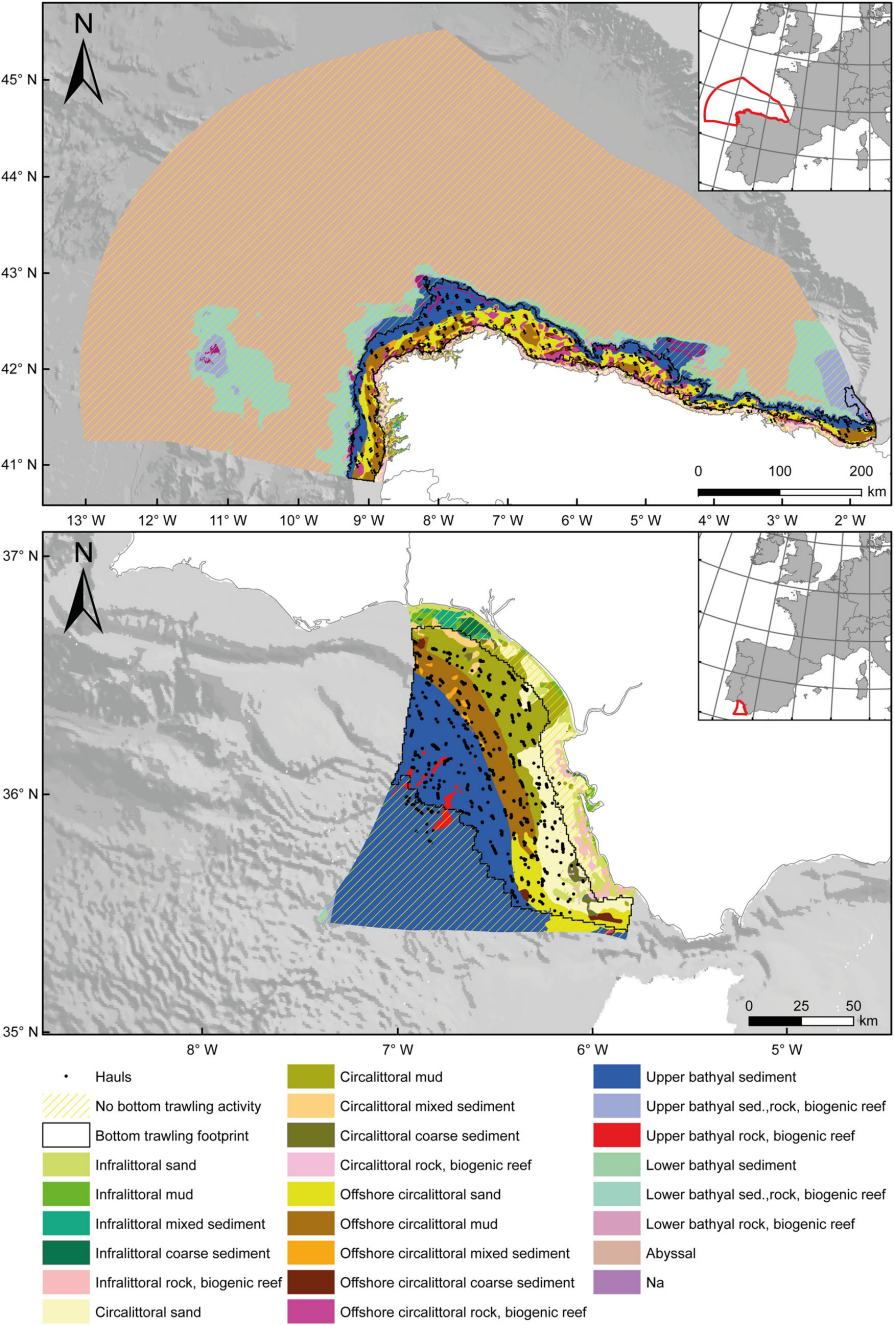
**Figure 1** Distribution of trawler hauls and broad habitat types based on EUSeaMap data (EMODnet, 2021) in the study areas in the (top) North Atlantic and (bottom) Gulf of Cadiz demarcation (yellow hatching, no bottom trawling activity 2016–2022; black outline, bottom trawling activity 2016–2022; black points, locations of biological database hauls).

The study covered the Spanish Atlantic waters, including the North Atlantic and Gulf of Cadiz demarcations (Figure 1), which are the Spanish MSFD assessment subdivisions. The habitat component (Figure 1) was based on the distribution and extent of the BHTs in the study area (EMODnet, 2021). The BHTs represented broadscale seabed habitat units derived from EUSeaMap 2021 produced by the EMODnet Seabed Habitats consortium. The map combines multiple environmental drivers, including water depth, seabed substrate, light availability, and biogeographic zones, that are used to predict habitat distribution at a broad scale. This approach is in line with the European Commission Decision (2017/848/E.U.), which outlines the scale of assessment for Descriptor 6 and was used to assess the relevant criteria of Descriptor 6, namely, physical disturbance and its adverse effects on benthic habitats (D6C2–D6C3) and habitat condition (D6C5). The coverage of each habitat type within the study area and the corresponding trawled area are shown in Figure 1 and Appendices S3 and S4.

The assessment focused on BHTs subjected to trawling effort; habitats not considered trawlable under the Spanish regulatory framework (e.g., abyssal or rocky habitats) were excluded. Any trawling effort values observed over rocky habitats are expected to be negligible and primarily reflect uncertainty in habitat mapping or the spatial resolution of the SAR dataset. Only BHTs that were biologically sampled sufficiently across the pressure gradient were included to fit statistically robust and ecologically interpretable generalized additive models (GAMs). In the North Atlantic demarcation, four BHTs (upper bathyal sediments, offshore circalittoral sand, offshore circalittoral mud, and offshore circalittoral mixed sediment) were analyzed (Figure 1). These covered approximately 82% of the demarcation's trawling footprint. This coverage increased to ∼95% when areas mapped as rocky habitats were excluded (Appendix S3). Apparent trawling effort over rocky areas is likely due to uncertainties in EMODnet habitat mapping or to the spatial resolution of the SAR grid, rather than real fishing effort. In the Gulf of Cadiz, only one BHT (upper bathyal sediment) was analyzed (Figure 1). This limitation was primarily due to the absence of reference areas (areas with no or low trawling effort, necessary for the SoS application) across most of the area (Figure 1; Appendix S4). Consequently, the assessment covered around 35% of the total trawling footprint in this area (Appendix S4).

### Bottom‐trawling pressure

The logbook and vessel monitoring systems (VMS) data from 2009 to 2022 were provided by the Fisheries Secretary, under the Ministry of Agriculture, Fisheries, and Food. These datasets were combined to determine the spatial dynamics of bottom‐contact fishing intensity from mobile gears (otter trawl and pair trawl) following the methods outlined in Fernandez‐Arcaya et al. (2024) and references therein. The swept area was calculated as the product of fishing hours, average fishing speed, and gear width. Gear width, expressed as surface and subsurface bottom contact, was estimated based on vessel length or engine power (kilowatts), following Eigaard et al. (2016), and refined with International Council for the Exploration of the Sea (ICES) expert input. Fishing intensity was expressed as the SAR in each grid cell estimated annually on a regular grid cell size of 0.05 × 0.05 decimal degrees (C‐squares) (Hintzen et al., 2012; ICES, 2019; Rees, 2003). The SAR values represented the theoretical number of times the total area of the grid cell would have been swept, assuming the fishing effort was evenly distributed within each cell (ICES, 2019).

### Benthic data collection

Benthic data were collected from the DEMERSALES and ARSA surveys, standardized bottom trawl surveys coordinated by the International Bottom Trawling Survey Working Group of the ICES. These surveys were conducted in the study areas from 2013 to 2022. Both surveys followed a random stratified sampling by depth, adhering to the standard IBTS methodology for northeastern Atlantic surveys (ICES, 2017). Each haul lasted 30 min and was conducted at a speed of 3 knots with an otter trawl gear (baca 44/60) with a 20‐mm mesh cod‐end liner. Detailed methodologies on sampling design, vessels, gears, and hauls are in ICES (2017).

For each haul, benthic species were identified, counted, and weighed. Biological traits related to size, longevity, motility, attachment, benthic position, flexibility, fragility, and feeding habitat were assigned to these species to apply the benthos sensitivity index to trawling operations (BESITO) (González‐Irusta et al., 2018). This index categorizes species according to their sensitivity to trawling based on biological traits analyses scores range from 1 (*very low sensitivity to trawling*) to 5 (*very high sensitivity*). The BESITO matrix excludes highly mobile invertebrate species with weak association with benthic habitats and covers up to 99% of the total biomass within the dataset evaluated.

To link each biological haul with the trawling pressure, trawling effort was calculated as the mean SAR for the 4 years preceding the sampling year plus the year of biological sampling. For instance, biological samples collected in 2013 were linked to the mean fishing intensity from 2009 to 2013. To ensure consistency between the available trawling effort data (2009‐2022) and biological data, the haul distribution was analyzed from 2013 to 2022.

The biological dataset included 23,146 records from DEMERSALES surveys, which covered 1304 sampling stations at depths ranging from 34 to 910 m, and 16,529 species records from ARSA surveys, spanning 762 sampling locations at depths of 18–863 m. Across these stations, 162 and 274 distinct benthic species were recorded in DEMERSALES and ARSA surveys, respectively. These datasets comprehensively cover the most extensive BHTs affected by trawling in the study area (Figure 1; Appendices S3 & S4).

### Environmental benthic status

To assess the environmental status of benthic habitats, we applied the SoS indicator (Serrano et al., 2022), which estimates the proportion of sentinel species as a proxy for benthic environmental status. This approach integrates benthic data from each BHT with fishing intensity. The process begins by identifying a list of sentinel species, which are the most sensitive taxa to the studied pressure from a set of typical species, those that are frequently present in the natural habitat. Typical species are selected based on their contribution to intrahabitat similarity between stations (similarity percentage procedure) (Clarke, 1993) and their occurrence frequency under reference or low pressure conditions. For bottom trawling, this initial set of typical species is filtered by prioritizing species with the highest BESITO scores (González‐Irusta et al., 2018). The higher the scores, the greater the sensitivity to trawling. Once the sentinel species list for each BHT is established, their relative biomass (proportion) within each sample is computed, capturing changes in the proportion of sentinel species across the fishing intensity gradient (SAR). Further methodological details on the generation of sentinel species lists are in Serrano et al. (2022).

The relationship between sentinel species proportion and trawling effort was modeled using GAMs, which produced pressure–state response curves for each habitat (Plaza‐Morlote et al., 2023; Serrano et al., 2022). The GAMs were selected to allow flexible fitting of nonlinear responses that may emerge due to complex ecological gradients. To enable standardized interpretation across BHTs, SoS values were rescaled from 0 to 1 by dividing each response curve by its maximum fitted value, which corresponds to the predicted proportion of sentinel species in untrawled conditions for each habitat (i.e., the GAM intercept). This normalization facilitates comparability of relative trends among habitats. However, it does not imply that SoS = 1 represents a community composed exclusively of sensitive species; rather, it indicates the average proportion of sensitive species in the absence of trawling pressure for that specific BHT. The resulting GAM‐derived curves were then applied to the spatial grid mean trawling intensity for each period to generate a spatial prediction of the sentinel species proportion in each grid cell. This prediction reflects the benthic status of each assessed BHT.

### Extent of adverse effects

We used three methods to set quality thresholds for assessing the extent of adverse effects on BHTs: distance to degradation (ICES, 2022a; Plaza‐Morlote et al., 2023), natural variation (Hiddink et al., 2023; ICES, 2022a; Landres et al., 1999; Rossberg et al., 2017), and detectable change (Hiddink et al., 2023; ICES, 2022a, 2022b). These methods differ in the way ecological thresholds are defined, each offering a unique perspective. These thresholds were expressed in state (proportion of sentinel species) and pressure (trawling intensity) terms to facilitate comparison.

The distance to degradation method (ICES, 2022a; Plaza‐Morlote et al., 2023) identifies the degradation point where the habitat has lost most of its quality and the loss of sentinel species starts to decelerate, causing the curve to flatten. This point is determined by locating where the slope of the response curve reaches a tangent of 45°, a method previously used to identify ecological tipping points (González‐Irusta & Wright, 2017). The quality threshold is then set at a defined distance from this point, adjusted for habitat sensitivity, with more sensitive habitats assigned greater distances. The quality threshold is calculated as a percentage of the distance between the curve's origin and the degradation point. To reflect varying habitat sensitivities, three distance values are explored (0.25 for habitats with sensitivity 4, 0.5 for sensitivity 3, and 0.75 for sensitivity 2). Sensitivity estimates are derived from pressure–state response curves (Elliot et al., 2018) (details in Serrano et al. [2022] and Plaza‐Morlote et al. [2023]). The distance to degradation method defines a state threshold, which is then applied to the spatial prediction of sentinel species proportion for each BHT. Cells with values above the threshold are classified as not adversely affected.

The natural variation approach defines a habitat as being in a good ecological state if it falls within the natural range of variability, typically expressed as the full observed range or a specific percentile, such as the 95th percentile (Hiddink et al., 2023; ICES, 2022a; Landres et al., 1999; Rossberg et al., 2017). Although some studies estimate this variability through interannual fluctuations at unaffected reference sites, we derived thresholds exclusively from long‐term data (2013–2022) collected at locations with zero trawling pressure, spanning the full spatial extent of each BHT. We assumed that spatially distributed data from zero‐pressure sites, when aggregated across multiple years and restricted to a single BHT (i.e., homogeneous sediment and depth range), can effectively capture the natural variability of the benthic community. Although spatial variation may not perfectly reflect temporal dynamics due to underlying environmental gradients, this method offers a pragmatic and ecologically relevant way to estimate thresholds in the absence of long‐term undisturbed time series.

Thresholds were calculated from the pressure–state GAM response curve with the lower 95% confidence interval of the sentinel species proportion at zero trawling pressure, which incorporated both uncertainty and natural fluctuations in the indicator. The state threshold derived from this approach was then applied to the spatial prediction of the proportion of sentinel species for each BHT, with cells above the threshold classified as not adversely affected.

The detectable change approach (Hiddink et al., 2023; ICES, 2022b) sets a pressure threshold for which the upper 95% confidence interval of sentinel species proportion intersects the lower 95% confidence interval at the lowest pressure level (ICES, 2022b)—in our study SAR =  0. We applied this threshold to trawling intensity spatial maps, masked by the extent of each BHT. Grid cells with pressure values below this threshold were classified as not adversely affected. This method aligns closely with the natural variation approach (Hiddink et al., 2023; ICES, 2022a) because the confidence intervals for nontrawling conditions capture the natural variability in sentinel species proportion at undisturbed sampling points.

### Indicator evaluation

For the indicator evaluation, the response variable (the SoS indicator) was predicted spatially based on an explanatory variable (trawling effort for the study period) with a correlative approach (GAMs). In essence, we employed a simplified species distribution model (Elith & Leathwick, 2009) involving only one variable to assess the status of benthic habitats. The prediction capacity of this method was evaluated using cross‐validation, a widely used technique for evaluating this kind of models in marine ecosystems (ICES, 2021). This method involved randomly selecting spatial blocks as training and testing data. The spatial blocks were generated using the cv_spatial function from the block CV R package (Valavi et al., 2019). Block size was determined by spatial autocorrelation in the explanatory variables. This ensured the spatial independence of each block. For the North Atlantic, 10 blocks were created, and five blocks were used for the smaller South Atlantic area. The size of the sampling hexagons was 46 km for the North Atlantic and 30 km for the South Atlantic. From each set of blocks, 60% were randomly selected as training data for model fitting, and 40% were used as test data to evaluate the model's predictions. The correlation between predicted and observed data was assessed using Spearman's rank correlation. This process was repeated 10 times for each demarcation and habitat type.

## RESULTS

### Bottom‐trawling intensity and footprint by habitat type

Bottom trawling was widespread across the Atlantic continental shelf of Spain; a few areas had <1 trawling event per year (Appendix S5). Grid cell trawling intensities (SAR) from 1 to 5 per year was common in larger regions, with hotspots in the eastern upper slope of the North Atlantic demarcation (SAR >10) and along the Gulf of Cadiz, where intensities exceeded 20 events per year. Trawling activity was mainly concentrated at depths <400 m. The highest intensities were from 200 to 400 m in the North Atlantic and from 20 to 200 m in the Gulf of Cadiz.

The bathymetric pattern of trawling led to significant differences in the swept area between regions. Northern Spain's narrow continental shelf had only 7.8% of its area affected by trawling, whereas the broader Gulf of Cadiz shelf had up to 53% affected.

Spatiotemporal analyses revealed that although the overall distribution and extent of the trawling footprint remained largely stable between the two periods, there was a reduction in trawling intensity (Appendices S3 & S4). Both regions exhibited significant trawling pressure on soft sediment habitats. Offshore circalittoral mud, offshore circalittoral sand, offshore circalittoral mixed sediment, and offshore circalittoral coarse sediment were the most heavily affected areas. In the Gulf of Cadiz, increased footprints also occurred in circalittoral mixed sediment and mud habitats. In northern Spain, the highest trawling intensities were found in offshore circalittoral mixed sediment, offshore circalittoral mud, upper bathyal sediment, and offshore circalittoral sand, and mean SAR values ranged from 2 to 5 events per year. In the Gulf of Cadiz, the most heavily trawled habitats included offshore circalittoral mixed sediment, offshore circalittoral mud, circalittoral mud, and circalittoral mixed sediment, with mean SAR values between 8.2 and 20.6 events per year. Many offshore circalittoral and circalittoral habitats in the Gulf of Cadiz were heavily trawled. The percentage of habitat area affected by trawling ranged from 67.1% to 100% (Appendix S4).

### Environmental benthic status concerning trawling

The sentinel species list differed between BHTs, and there was minimal overlap in the sentinel species set between the two management areas. Some species were, however, recurrently present in several BHTs (e.g., sea pen [*Funiculina quadrangularis*] and the holothuroid *Parastichopus regalis*) (Table 1). Despite this, sentinel species were generally specific to each BHT, with most species being designated as sentinel species for only one BHT. An exception was in the offshore circalittoral mud and offshore circalittoral sand habitats from the North Atlantic demarcation, where there was a higher degree of similarity in sentinel species composition.

**TABLE 1:** Final sentinel species^a^ set, BESITO (benthos sensitivity index to trawling operations) scores, and number of low‐pressure^b^ trawler hauls per broad habitat type (BHT) in the North Atlantic and Gulf of Cádiz demarcations.

BHT	SAR	HAULS	BESITO	Sentinel species
North Atlantic				
UBatSed	≤0.1	48	5	*Acanella arbuscula*, *Asconema setubalense*, *Phakellia ventilabrum*, *Pheronema carpenteri*
			4	*Funiculina quadrangularis*, *Hymenodiscus coronata*, *Kophobelemnon stelliferum*
			3	*Phormosoma placenta*, *Gracilechinus acutus*, *Parastichopus tremulus*, *Parastichopus regalis*, *Araeosoma fenestratum*, *Nymphaster arenatus*
OfCirSa	≤0.1	29	5	*Phakellia ventilabrum*
			4	*Corella parallelogramma*, *Funiculina quadrangularis*
			3	*Gracilechinus acutus*, *Ophiothrix fragilis*, *Parastichopus regalis*, *Spatangus purpureus*, *Anseropoda placenta*, *Echinus melo*, *Nymphaster arenatus*
OfCirMu	≤0.65	19	5	*Phakellia ventilabrum*
			4	*Corella parallelogramma*, *Pteroeides spinosum*
			3	*Gracilechinus acutus*, *Parastichopus regalis*, *Ophiothrix fragilis*, *Pennatula phosphorea*, *Anseropoda placenta*, *Leptometra celtica*, *Lytocarpia myriophyllum*
OfCirMs	≤0.33	4	4	*Funiculina quadrangularis*
			3	*Gracilechinus acutus*, *Parastichopus regalis, Spatangus purpureus*, *Actinauge richardi*, *Ophiura ophiura*
			2	*Marthasterias glacialis*
Gulf of Cadiz				
UBatSed	≤0.1	49	5	*Asconema setubalense*, *Geodia* sp.
			4	*Hymenodiscus coronata*, *Thenea muricata*,
			3	*Parastichopus tremulus*, *Parastichopus regalis*, *Hormathia alba*, *Flabellum chuni*, *Diphasia margareta*

Abbreviations: OfCirMs, offshore circalittoral mixed sediment; OfCirMu, offshore circalittoral mud; OfCirSa, offshore circalittoral sand; UBatSed, upper bathyal sediment.

^a^Species typical of the habitat and sensitive to the pressure.

^b^The swept area ratio (SAR) thresholds used to obtain the list of sentinel species are based on reference and low‐pressure conditions. The number of hauls reflects data availability and the need to ensure species representativeness within each BHT and area.

As conceptually expected, the proportion of sentinel species that responded to trawling showed a strongly negative and statistically significant correlation (*p* < 0.001) with trawling in all the BHT datasets (Appendix S6). However, as can be seen in Appendix S6, the intensity of the response varied among BHTs, reflecting differences in habitat sensitivity to trawling effort. In general, habitats that hosted species from highly sensitive groups, such as the upper bathyal sediment of the Gulf of Cadiz, demonstrated a pronounced decline in sentinel species proportions. In contrast, more resilient habitats with less sensitive species groups, such as OfCirMs, exhibited a less steep decline. Importantly, the sensitivity of a habitat under reference (undisturbed) conditions (linked to its inertia of disturbance [van Denderen et al., 2015]) did not always correspond directly to the response strength observed under trawling pressure. Variations in the contribution of each species to total biomass and differences in real sensitivity between species with the same BESITO value may also have affected the strength and shape of the observed response (e.g., offshore circalittoral mud; Appendix S6). To support model transparency and allow visualization of the raw model fit, full GAM plots, including residuals and confidence intervals prior to normalization, are provided in Appendix S7.

Although the response curves generally showed the expected negative relationship between the proportion of sentinel species and trawling effort, two curves (offshore circalittoral mud and offshore circalittoral mixed sediment) displayed an unexpected positive trend after reaching a minimum at the highest trawling effort values (Appendix S6). This increase is an artifact of edge effects in the GAMs, which can bias the shape of the fitted response curves when data are sparce (Hastie & Tibshirani, 1990), particularly at high trawling effort values (SAR >7 per year). To avoid this bias, the response curves were corrected by giving them the lowest value of the sentinel species proportion of the dataset for SAR <7 per year and its associated error for all proportions above 7 SAR per year (Appendix S6). This action was based on the assumption that the proportion of sentinel species does not increase at high trawling intensities and that the observed pattern is driven by model artifacts.

**FIGURE 2:**
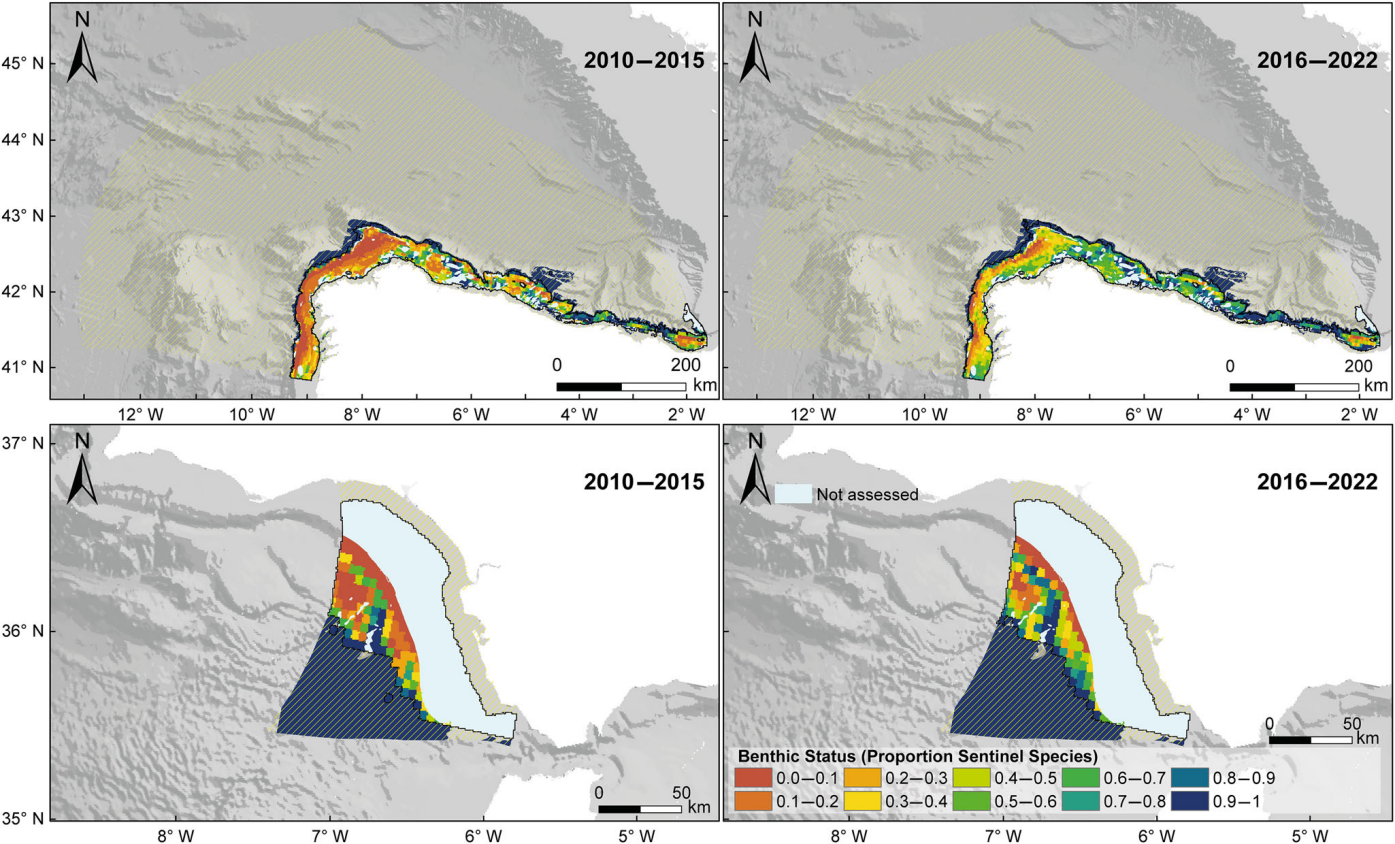
**Figure 2** Predicted proportion of sentinel species (species typical of the habitat and sensitive to the pressure) relative to the mean swept area ratio values for two periods in (top) North Atlantic demarcation and (bottom) Gulf of Cadiz study areas (blue shading, broad habitat types unassessed but subject to bottom trawling; yellow hatching, no bottom trawling activity; black outline, bottom trawling activity).

The predicted benthic community status, represented by the predicted proportion of sentinel species, is shown in Figure 2, with model uncertainty detailed in Appendix S8. A sentinel species proportion of 1 indicated an unaffected benthic status with no trawling activity (SAR = 0 per year), whereas lower values reflected increasing levels of impact. Across most study areas, the sentinel species proportion ranged from 0.2 to 1. The lowest values (<0.2) were concentrated in areas of the upper bathyal sediment and offshore circalittoral sand habitats from the eastern continental upper slope of the North Atlantic demarcation and in the shallower areas of the Gulf of Cadiz. These highly affected areas aligned with grid cells that supported the highest fishing intensity in the most sensitive BHT (upper bathyal sediment), as well as in less sensitive BHTs, such as offshore circalittoral mud (Figures 1 & 2; Appendices S5 & S9).

Mean benthic status values for each assessed BHT and period (Appendix S10) indicated a general improvement in benthic status in the most recent period compared with 2010–2015 across all analyzed BHTs. When considering the total area of each BHT, mean values were as low as 0.23 across the assessed habitats; the highest value was 0.63 in the upper bathyal sediment habitat across both study areas. This pattern reflected the large number of untrawled cells in this BHT. However, when focusing only on trawled areas, the mean benthic status value for upper bathyal sediment dropped by 0.41, indicating a marked decline in benthic condition associated with trawling and highlighting the high sensitivity of this habitat.

### Extent of adverse impacts on trawling

**FIGURE 3:**
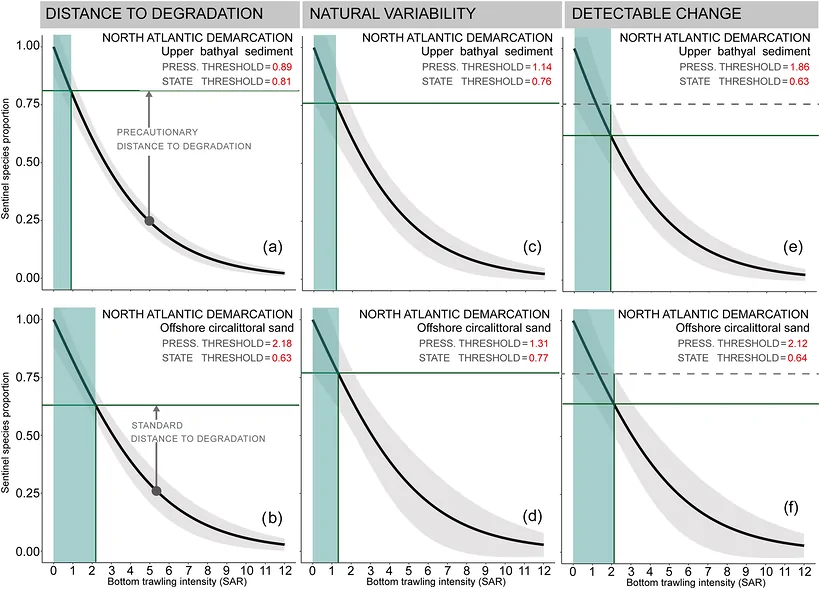
**Figure 3** Derivation of habitat quality thresholds for the upper bathyal sediment and offshore circalittoral sand habitat types in study sites in the North Atlantic based on three approaches: (a, b) distance to degradation (shading, SE), (c, d) natural variability (shading, 95% confidence interval), and (e, f) detectable change (shading, 95% confidence interval) (solid green lines, thresholds distinguishing good from degraded habitat quality including pressure [press.] and state thresholds; gray lines and points, additional reference values used in threshold derivation).

**FIGURE 4:**
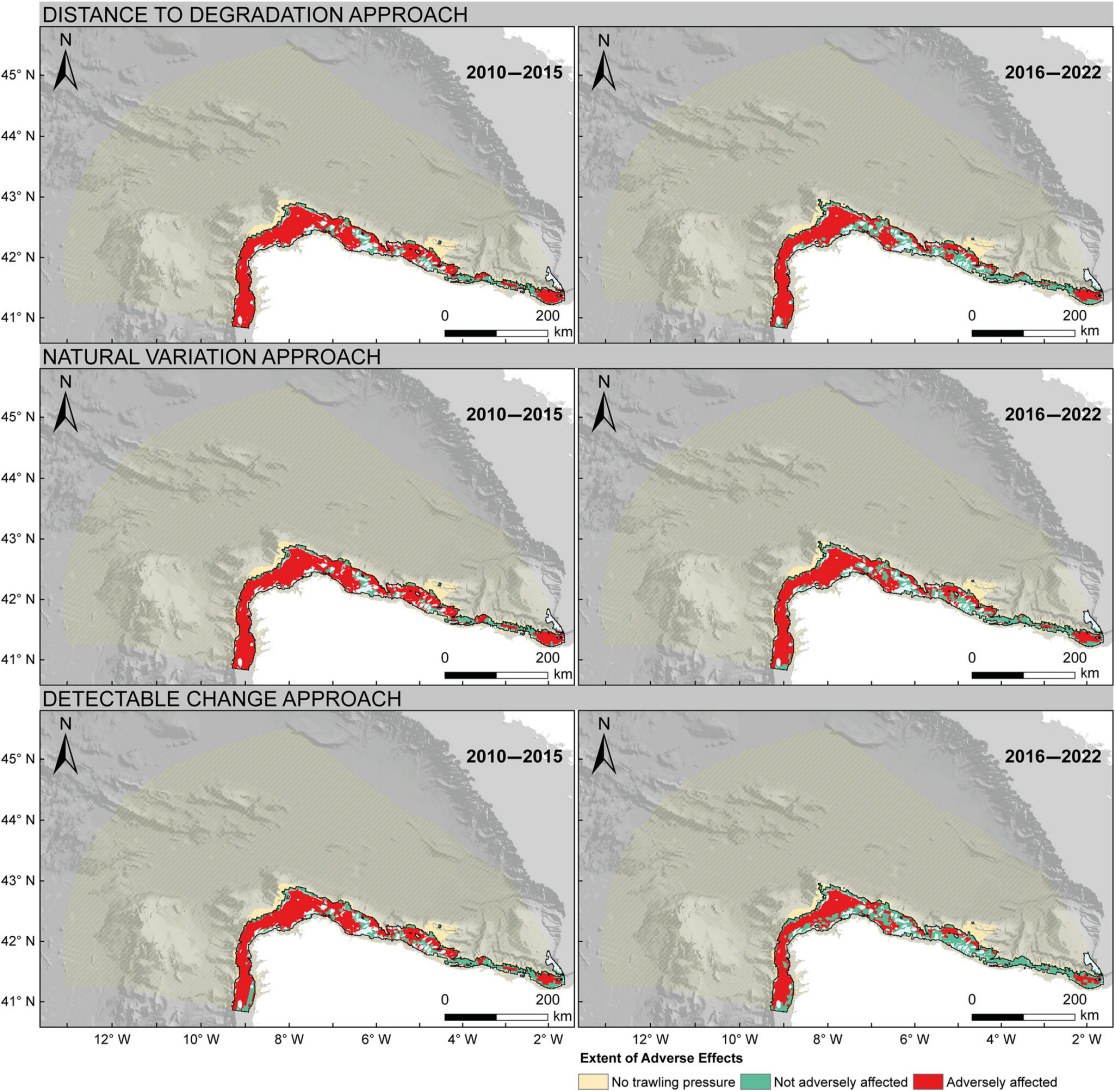
**Figure 4** The extent of adverse effects of bottom trawling across broad habitat types in the North Atlantic based on the mean swept area ratio values for (left) 2010–2015 and (right) 2016–2022–and three thresholds: (top) distance to degradation threshold, (middle) natural variation threshold, and (bottom) detectable change threshold.

**FIGURE 5:**
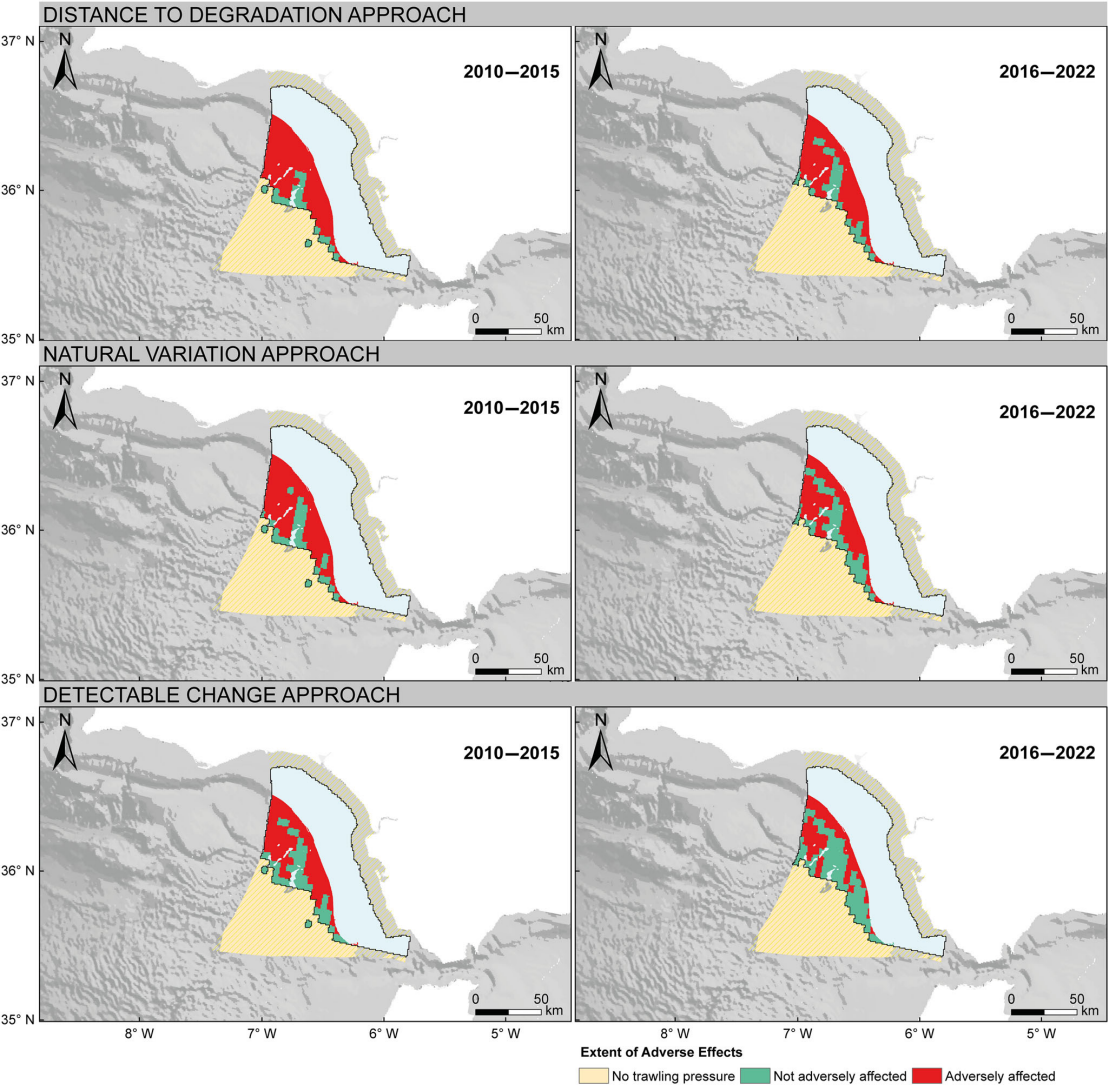
**Figure 5** The extent of adverse effects of bottom trawling across broad habitat types in the Gulf of Cadiz based on the mean swept area ratio values for (left) 2010–2015 and (right) 2016–2022 and three thresholds: (top) distance to degradation threshold, (middle) natural variation threshold, and (bottom) detectable change threshold (light blue, broad habitat types not analyzed due to absence of reference areas or insufficient data).

**FIGURE 6:**
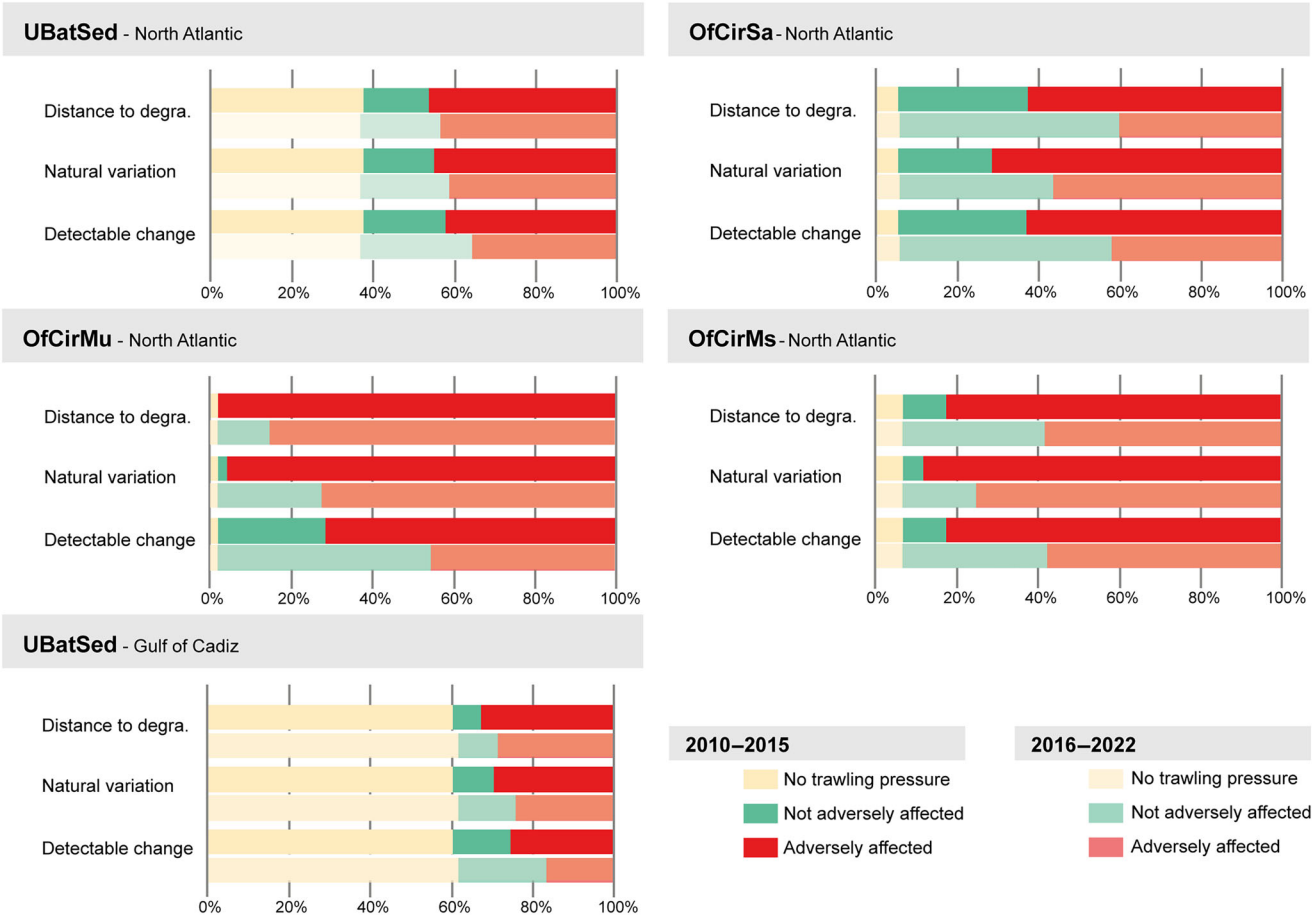
**Figure 6** Percentage of habitat extent adversely affected by trawling for each analyzed broad habitat type (UBatSed, OfCirSa, OfCirMu, OfCirMs). Results are shown for three quality thresholds and two periods (2010–2015 vs. 2016–2022) in the studied areas in the North Atlantic and Gulf of Cadiz.

The three approaches used to establish quality thresholds for each BHT allowed the classification of seabed areas as adversely affected by trawling (Figure 3; Appendices S11 & S12). The detectable change method generally produced more permissive threshold values than the other two methods, leading to noticeable differences in the extent of affected areas. The natural variation approach was the most conservative, whereas the detectable change method was the least restrictive (Figures 3, 4, 5, 6; Appendices S11–S13). Despite these variations, all methods showed statistically significant positive correlations between the grid cell values (*p* < 0.05).

In the North Atlantic (2010–2015), approximately 7.8% (∼24,510 km^2^) of the seabed was trawled (Appendix S13). The percentage of adversely affected areas ranged from 4.2% to 4.9%, depending on the method, with 1.4% (∼4445.7 km^2^) of the seabed unassessed due to data limitations. By 2016–2022, trawled seabed remained at 7.9%, with affected areas varying from 3.13% to 4.09%. An estimated 1.43% (∼4470 km^2^) of the seabed remained unassessed during this period. The most affected regions were concentrated along the Galician coast (Figure 4).

Despite methodological differences, all approaches consistently identified upper bathyal sediment and offshore circalittoral sand as the most extensively affected BHTs in terms of total area, given their larger spatial extent. However, the most impacted habitats in terms of the proportion of area classified as adversely affected were offshore circalittoral mud and offshore circalittoral mixed sediment. Discrepancies were observed in the case of offshore circalittoral mud, especially for 2016–2022, for which the distance to degradation and natural variation methods estimated a significantly greater affected extent than the detectable change method (Figure 6; Appendix S13). Despite these impacts, 92.2% of the North Atlantic seabed remained unaffected by bottom trawling, with untrawled areas primarily found in offshore deep waters (>800 m depth) and in shallower waters below 100 m. Such areas are typically excluded from trawling due to regulatory restrictions (EU 2016/2336; Real Decreto 502/2022; Real Decreto 1441/1999) (Figures 4 & 6; Appendix S13).

In the Gulf of Cádiz, 54.1% (∼7566.8 km^2^) of the seabed was trawled in 2010–2015. The estimated affected areas ranged from 12.3% to 15.75%, with 35.01% (∼4896.9 km^2^) of the seabed unassessed due to the lack of reference areas and benthic samples. By 2016–2022, the trawled seabed was 53.2%, with adversely affected areas ranging from 8.0% to 13.62%. A total of 34.88% (∼4877.77 km^2^) of the seabed remained unassessed due to data limitations. A large portion of the seabed (up to 46%) remained untrawled, particularly in deep waters (>800 m) and areas with trawling restrictions (Figures 5 & 6; Appendix S13).

### Evaluation metrics

Of the five BHTs evaluated, the upper bathyal sediment in both study regions and the offshore circalittoral mixed sediment were positively and significantly correlated in all the evaluation iterations, whereas offshore circalittoral sand and offshore circalittoral mud were nonsignificantly correlated in two and three iterations, respectively (Appendix S14). These habitats also had the lowest average rho values between predicted and observed SoS values, with a mean (SD) of 0.36 (0.16) for offshore circalittoral sand and 0.25 (0.14) for offshore circalittoral mud. Offshore circalittoral mixed sediment showed the strongest correlation between predicted and observed SoS values (mean [SD] rho of 0.66 [0.13]). Finally, the upper bathyal sediment habitats in both study regions had the most consistent correlation between predicted and observed values across iterations, as shown by the lower SD values and relatively high average rho values.

## DISCUSSION

Assessing benthic habitat quality in areas exposed to bottom trawling requires a robust understanding of the natural seabed condition, impact levels, and pressure–state relationships. Over the past two decades, substantial progress has been made to characterize fishing impacts and recovery patterns (e.g., Collie et al., 2000; Kaiser et al., 2006; Pitcher et al., 2022; van Denderen et al., 2015) and to develop and test benthic indicators across regions (e.g., Serrano et al., 2022; Smith et al., 2023; van Denderen et al., 2024). Nevertheless, inconsistencies in methodologies and indicators, and the absence of standardized quality thresholds, pose major challenges globally, especially within the EU's MSFD framework. We provided the first quantitative assessment of bottom‐trawling impacts on sedimentary habitats in Atlantic waters based on the SoS indicator (Serrano et al., 2022). By establishing scientifically grounded quality thresholds, we defined trawling impacts for each BHT.

The SoS indicator, recognized for its sensitivity to trawling pressure (ICES, 2022b; Serrano et al., 2022; van Denderen et al., 2024), demonstrated a strong and statistically significant negative correlation (*p* < 0.001) between the proportion of sentinel species and trawling intensity across all BHT datasets. Its effectiveness stems from the definition of sensitive taxa based on a sentinel species list derived from the BESITO index (Gonzalez‐Irusta et al., 2018). The accuracy of the results, particularly in predicting sentinel species proportions with GAM models, was validated through cross‐validation following ICES recommendations for predictive distribution models (ICES, 2021). Spearman's rho values, ranging from 0.3 to 0.6, indicated moderate to strong correlations and were comparable to (or stronger than) those achieved in previous predictive modeling studies (Arronte et al., 2024; Gonzalez‐Irusta & Wright, 2016, 2017; Lelievre et al., 2014). These results, obtained through independent biological validation, support the robustness of the SoS indicator, despite natural environmental heterogeneity, ensuring it is a reliable tool for detecting shifts in predictive benthic status, as suggested by previous studies (ICES, 2022b; Serrano et al., 2022; van Denderen et al., 2024). Additionally, the SoS indicator is a recognized common indicator under OSPAR (BH1) with a standardized methodology (Plaza‐Morlote et al., 2023; Serrano et al., 2022) that ensures its applicability across regions and policy frameworks (ICES, 2022b; Serrano et al., 2022; van Denderen et al., 2024).

Defining ecological thresholds for benthic indicators remains one of the key challenges for assessing environmental status under the MSFD (Elliott et al., 2018; ICES, 2022a; Lambert et al., 2017). We applied and compared three complementary approaches: natural variation, distance to degradation, and detectable change. Each offers useful perspectives for setting thresholds, despite their inherent limitations (Hiddink et al., 2023; ICES, 2022a; Plaza‐Morlote et al., 2023). The thresholds estimated with the natural variation method, based on the range of values observed under no trawling pressure, would ideally be derived from long‐term, consistent, annual sampling over at least 10 consecutive years of benthos in minimally affected areas (ICES, 2025). However, such datasets are scarce for undisturbed habitats (Sibly et al., 2007), so we derived these thresholds from modeled long‐term trends rather than from fixed historical baselines. The limited availability of untrawled reference samples adds uncertainty, particularly in offshore circalittoral mud and offshore circalittoral sand, where broad confidence intervals complicate threshold determination. This issue is especially relevant in habitats characterized by high spatial heterogeneity. For instance, in upper bathyal sediment, the wide range of depths and substrates contributes to natural variation in the proportion of sensitive species, reflecting the patchy nature of the habitat. As noted by Hiddink et al. (2023), this approach aligns conceptually with the idea that low‐pressure confidence intervals reflect natural variability, though careful interpretation is required in heterogeneous environments.

The detectable change method, thoroughly discussed by Hiddink et al. (2023), defines thresholds based on the minimum level of change that can be statistically detected. Although this is a transparent and objective approach, its main limitation is that it reflects the statistical power of the dataset rather than ecological degradation. As Hiddink et al. (2023) point out, “detectability is only a function of the power to detect effects rather than a definition of ecological degradation of state.” Thus, a lack of detectability may simply indicate a lack of statistical power, whereas a high level of detectability could identify changes that are not ecologically meaningful. Moreover, detectability is likely to decrease with increasing natural variability, making this method particularly sensitive to the inherent variability of benthic systems. As highlighted in our results, the detectable change method tended to produce lower estimates of affected areas.

In contrast to previous methods based on reference conditions (i.e., start of the response curve), the distance to degradation method identifies inflection points at the end of the pressure–state relationship, offering an ecologically meaningful interpretation of when habitat condition begins to decline significantly (Plaza‐Morlote et al., 2023). One of its main advantages is that it does not rely on confidence intervals and is thus less affected by limited replication in reference areas. However, it has been applied only with the SoS indicator (ICES, 2022a) and may not be appropriate in cases where the indicator responds linearly to pressure (e.g., very resilient habitats that never reach the degradation point, unlikely for trawling).

Although methodological differences exist, all three approaches produced consistent patterns of relative risk across BHTs. Natural variation and distance to degradation showed similar results, with the strongest correlation observed between them (*r* = 0.86). The detectable change method yielded lower impact estimates, particularly concerning sensitive habitats like upper bathyal sediment. Nonetheless, all methods consistently identified the same habitats as at high risk, supporting the robustness of the assessment regardless of the threshold approach used. Ultimately, although each method had its limitations, their combined application enhanced the reliability of benthic impact assessments. Together, they provided a more comprehensive understanding of ecosystem condition, balancing ecological meaning with statistical rigor. This multimethod approach contributes to a sound and transparent framework for evaluating seafloor status under the MSFD and supports the development of regionally adapted conservation and management strategies.

The extent of adverse effects from bottom trawling varied across regions, assessment periods, and threshold methodologies (Figure 6; Appendix S13). In the North Atlantic management area, despite differences in estimated thresholds and evaluation periods, all approaches consistently identified upper bathyal sediment and offshore circalittoral sand as the most extensively affected BHTs. This pattern was likely driven by the large spatial extents of these habitats and their high cumulative trawling footprint, as previously reported in the region (Fernandez‐Arcaya et al., 2024). In particular, upper bathyal sediment stood out not only due to its extent and exposure but also due to the presence of highly sensitive species (de la Torriente et al., 2022; Grinyó et al., 2020; Serrano et al., 2022), such as *Acanella arbuscula*, *Asconema setubalense*, and *Phakellia ventilabrum*, which are recognized indicators of vulnerable marine ecosystems (Clark et al., 2016).

In terms of relative impact, offshore circalittoral mud and offshore circalittoral mixed sediment presented the highest proportion of their total area affected, which placed them among the most impacted habitats in the North Atlantic. The scarcity of untrawled reference areas hampers robust assessment and further compromises their ecological resilience. These habitats overlap with important core fishing grounds for the Spanish fleet targeting key demersal species, such as hake, anglerfish, and Nephrops (Punzón et al., 2010). The patterns we found are consistent with findings across Europe, where mud and mixed sediments have been repeatedly highlighted as among the most intensively trawled habitats. For instance, in the Bay of Biscay, over 99% of offshore circalittoral mud and mixed sediment show evidence of fishing activity (Fernández‐Arcaya et al., 2024), and in the North Sea, similar habitats are trawled on average 3–4.2 times per year (Rijnsdorp et al., 2020). The Gulf of Cádiz exhibited considerably higher overall trawling pressure, with over 50% of the seabed trawled in both assessment periods, substantially more than the 7.8%–7.9% recorded in the North Atlantic. As a result, the extent of adversely affected habitats in Cádiz was greater, with detailed assessments limited to upper bathyal sediment due to the lack of untrawled reference areas in other BHTs.

Nonetheless, the strong correlation found between trawling intensity (SAR) and the SoS indicator suggests that SAR could serve as a practical proxy for estimating ecosystem status in data‐limited BHTs. Based on trawling intensity and footprint data, it is likely that offshore circalittoral mixed sediment, offshore circalittoral mud, circalittoral mud, and circalittoral mixed sediment habitats in the Gulf of Cádiz are among the most severely affected. These are the same BHTs identified by Eigaard et al. (2017) as being most frequently and heavily trawled across European seas and the Mediterranean, reinforcing the consistency and broader applicability of our findings. Despite the intensity of bottom trawling, a significant proportion of the seabed in both regions remained untrawled. This is largely due to legal restrictions that prohibit bottom trawling below certain depths or in specific areas (e.g., EU Regulation 2016/2336; Real Decreto 1441/1999; Real Decreto 502/2022; Real Decreto 632/1993).

Although the spatial distribution of the trawl footprint is stable in different regions (Amoroso et al., 2018; Hilborn et al., 2023), we found a significant reduction in trawling intensity across all BHTs, as noted by Fernandez‐Arcaya et al. (2024) for our study area. This reduction is likely driven by various factors, such as a decrease in fleet size, rising fuel costs (Prellezo et al., 2020), spatial management regulations (E.C. 2166/2005), and competition for fishing grounds (Punzón et al., 2010, 2016). This decline in trawling intensity suggests that many habitats may be on a path to achieving GES, provided these trends are maintained. However, large habitat areas remain at risk, particularly in the Gulf of Cádiz, where the extent of disturbance far exceeds that observed in northern Spain. None of the BHTs we assessed met the 25% extent threshold for adversely affected areas, which was formally adopted under the MSFD as a benchmark for assessing benthic ecosystem status, although it is not officially regulated. If the current trend persists, significant improvements may be observed, but additional measures may be necessary for the most affected habitats.

Our assessment approach has several limitations. First, seabed habitat classifications are based on BHT predictive mapping (EMODnet, 2021), which introduces spatial uncertainties (Vasquez et al., 2021). Future work at the community level may further refine these assessments and reduce mismatches between mapped habitats and observed benthic communities. Second, estimates focused on the Spanish fleet, potentially underestimating total pressure in certain areas for other countries’ fleets. Third, our assessments could have been improved in shallow waters by integrating inshore VMS (Murillas‐Maza et al., 2023; Silvestrini et al., 2024). Fourth, refining spatial resolution of trawling data would have enhanced accuracy, particularly in complex geomorphologies (Fernandez‐Arcaya et al., 2024). Firth, we did not account for additional environmental drivers that may influence benthic communities, such as depth, currents, primary productivity, or temperature. Incorporating these variables in future models could improve predictive accuracy and better capture habitat‐specific variability. Sixth, reference samples in Cádiz needed to validate impact estimates across all BHTs were lacking. Addressing these limitations would significantly enhance assessment accuracy and reduce uncertainty. In addition, because no single indicator can fully meet all MSFD requirements, combining complementary indicators is necessary to properly assess the state of benthic habitats under Descriptor 6 (ICES, 2022b).

The spatial assessment of trawling impacts we presented provides critical insights for conservation initiatives and sustainable fisheries management. By identifying high‐impact areas, it supports adaptive management strategies that prioritize region‐specific conservation measures tailored to habitat sensitivities and regulatory contexts. Offering a robust, quantitative framework to assess seafloor integrity, this study directly contributes to MSFD objectives and narrows the gap between scientific knowledge and practical decision‐making by delivering tools ready for management application. Ultimately, it supports regionally adapted marine spatial planning and fisheries policies to strengthen the long‐term resilience of benthic ecosystems.

## AUTHOR CONTRIBUTIONS

**Maider Plaza‐Morlote**: Conceptualization; methodology; formal analyses; visualization; writing—original draft; writing—review and editing. **Ana García‐Alegre**: Conceptualization; methodology; formal analyses; visualization; writing—original draft; writing—review and editing. **Alberto Serrano**: Project administration; conceptualization; methodology; writing—review and editing. **Ulla Fernández‐Arcaya**: Formal analyses; writing—review and editing. **Antonio Punzón**: Data curation; formal analyses; writing—review and editing. **Ana Rodríguez‐Rúa**: Formal analyses; writing—review and editing. **Sara Roman**: Formal analyses; writing—review and editing. **Ana Magro**: Formal analysis; writing—review and editing. **Marina Delgado**: Data curation; writing—review and editing. **Juan Gil**: Data curation; writing—review and editing. **José Manuel González‐Irusta**: Conceptualization; supervision; methodology; formal analyses; writing—original draft; writing—review and editing.

## Supporting information



Supporting Information

Supporting Information

Supporting Information

Supporting Information

Supporting Information

Supporting Information

Supporting Information

## Data Availability

The data supporting this research are held by the Spanish Institute of Oceanography and, in accordance with institutional policy, cannot be uploaded to a public repository. However, they may be provided by the corresponding author upon request.
