# Supplementary material for: Tracking the impact of bottom trawling on benthic habitat status
Source: Conserv Biol. 2026 Apr 22;40(5):e70303. doi: 10.1111/cobi.70303 (PMC13613570; doi:10.1111/cobi.70303)
Supplement: Supplementary file 1 [file COBI-40-e70303-s002.docx]

**Appendices**

*Appendix S1. Schematic workflow of the methodology applied to assess benthic habitat status and the extent of adverse effects from bottom trawling. The workflow includes (1) input data layers, (2) generation of sentinel species lists, (3) pressure–state response curves, (4) environmental benthic status estimation, (5) application of quality threshold approaches, and (6) mapping of the extent of adverse effects. Only the distance-to-degradation threshold method is illustrated here as an example to facilitate understanding of the approach.*

Appendix S2. List of the Broad Habitats Types in the study areas and the abbreviations used.

| BHT | BHT abbreviation |
| --- | --- |
| Infralittoral sand | InfSa |
| Infralittoral mud | InfMu |
| Infralittoral mixed sediment | InfMs |
| Infralittoral coarse sediment | InfCs |
| Infralittoral rock and biogenic reef | InfRo |
| Circalittoral sand | CirSa |
| Circalittoral mud | CirMu |
| Circalittoral mixed sediment | CirMs |
| Circalittoral coarse sediment | CirCs |
| Circalittoral rock and biogenic reef | CirRo |
| Offshore circalittoral sand | OfCirSa |
| Offshore circalittoral mud | OfCirMu |
| Offshore circalittoral mixed sediment | OfCirMs |
| Offshore circalittoral coarse sediment | OfCirCs |
| Offshore circalittoral rock and biogenic reef | OfCirRo |
| Upper bathyal sediment | UBatSed |
| Upper bathyal sediment or rock and biogenic reef | UBatS/Ro |
| Upper bathyal rock and biogenic reed | UBatRo |
| Lower bathyal sediment | LBatSed |
| Lower bathyal sediment or rock and biogenic reef | LBatSeRo |
| Lower bathyal rock and biogenic reed | LBatRo |
| Abyssal | Abyss |
| Na | Na |

*Appendix S3. Overview of Benthic Habitat Types (BHT) in the maritime waters of the North Atlantic Demarcation. For each BHT, the table presents the total area (km²), trawled area (km²), percentage of BHT area affected by trawling, proportion of the total trawling footprint represented by the BHT, and both total and mean fishing intensity (SAR) supported by the BHT. Fishing activity (trawling footprint) was determined based on average SAR values for the two timeframes, considering SAR values > 0* **y^-1^***. See Supplementary Table 1 for habitat name abbreviations.*

| BHT | AREA (Km^2^) | AREA WITH TRAWLING (Km^2^) | | % HÁBITAT TRAWLED | | % HAB TO TRAW. FOOT | | TOTAL FI (SAR) | | MEAN FI (SAR) TOTAL HABITAT AREA | | MEAN FI (SAR) TRAWLED HABITAT AREA | |
| --- | --- | --- | --- | --- | --- | --- | --- | --- | --- | --- | --- | --- | --- |
|  |  | 2010-2015 | 2016-2022 | 2010-2015 | 2016-2022 | 2010-2015 | 2016-2022 | 2010-2015 | 2016-2022 | 2010-2015 | 2016-2022 | 2010-2015 | 2016-2022 |
| UBatSed | 13127.7 | 8174.6 | 8301.4 | 62.3 | 63.2 | 33.4 | 33.7 | 2596.0 | 1325.1 | 3.7 | 1.9 | 6.0 | 3.0 |
| OfCirSa | 7545.2 | 7145.7 | 7127.5 | 94.7 | 94.5 | 29.2 | 28.9 | 1412.1 | 784.9 | 3.5 | 1.9 | 3.7 | 2.0 |
| OfCirMu | 4220.5 | 4135.9 | 4136.2 | 98.0 | 98.0 | 16.9 | 16.8 | 1013.6 | 627.4 | 4.5 | 2.8 | 4.6 | 2.8 |
| OfCirRo | 2916.0 | 2282.8 | 2289.1 | 78.3 | 78.5 | 9.3 | 9.3 | 240.9 | 128.9 | 1.5 | 0.8 | 2.0 | 1.0 |
| OfCirCs | 1189.6 | 1006.2 | 1001.7 | 84.6 | 84.2 | 4.1 | 4.1 | 109.2 | 64.0 | 1.7 | 1.0 | 2.0 | 1.2 |
| OfCirMs | 648.4 | 605.2 | 605.2 | 93.3 | 93.3 | 2.5 | 2.5 | 163.0 | 83.9 | 4.6 | 2.4 | 5.0 | 2.6 |
| UBatRo | 1440.6 | 541.6 | 573.8 | 37.6 | 39.8 | 2.2 | 2.3 | 114.5 | 59.8 | 1.5 | 0.8 | 3.9 | 1.9 |
| UBatS/Ro | 4930.3 | 382.6 | 417.8 | 7.8 | 8.5 | 1.6 | 1.7 | 11.4 | 6.4 | 0.0 | 0.0 | 0.6 | 0.3 |
| Na | 68.2 | 59.6 | 59.6 | 87.3 | 87.3 | 0.2 | 0.2 | 5.3 | 3.0 | 1.5 | 0.8 | 1.6 | 0.9 |
| CirSa | 1611.8 | 55.9 | 56.3 | 3.5 | 3.5 | 0.2 | 0.2 | 4.8 | 3.6 | 0.1 | 0.0 | 1.8 | 1.4 |
| CirRo | 2362.4 | 42.8 | 42.4 | 1.8 | 1.8 | 0.2 | 0.2 | 2.9 | 2.0 | 0.0 | 0.0 | 1.4 | 0.9 |
| CirMu | 328.4 | 21.7 | 21.7 | 6.6 | 6.6 | 0.1 | 0.1 | 1.2 | 0.9 | 0.0 | 0.0 | 1.2 | 0.9 |
| CirCs | 497.7 | 4.9 | 4.7 | 1.0 | 0.9 | 0.0 | 0.0 | 0.0 | 0.0 | 0.0 | 0.0 | 0.0 | 0.0 |
| InfRo | 742.6 | 0.2 | 0.0 | 0.0 | 0.0 | 0.0 | 0.0 | 0.0 | 0.0 | 0.0 | 0.0 | 0.0 | 0.0 |
| InfSa | 388.2 | 0.2 | 0.2 | 0.0 | 0.1 | 0.0 | 0.0 | 0.0 | 0.0 | 0.0 | 0.0 | 0.0 | 0.0 |
| CirMs | 134.2 | 0.0 | 0.0 | 0.0 | 0.0 | 0.0 | 0.0 | 0.0 | 0.0 | 0.0 | 0.0 | 0.0 | 0.0 |
| InfCs | 45.0 | 0.0 | 0.0 | 0.0 | 0.0 | 0.0 | 0.0 | 0.0 | 0.0 | 0.0 | 0.0 | 0.0 | 0.0 |
| InfMs | 120.4 | 0.0 | 0.0 | 0.0 | 0.0 | 0.0 | 0.0 | 0.0 | 0.0 | 0.0 | 0.0 | 0.0 | 0.0 |
| InfMu | 146.1 | 0.0 | 0.0 | 0.0 | 0.0 | 0.0 | 0.0 | 0.0 | 0.0 | 0.0 | 0.0 | 0.0 | 0.0 |
| LBatRo | 493.9 | 0.0 | 0.0 | 0.0 | 0.0 | 0.0 | 0.0 | 0.0 | 0.0 | 0.0 | 0.0 | 0.0 | 0.0 |
| LBatSeRo | 19545.0 | 0.0 | 0.0 | 0.0 | 0.0 | 0.0 | 0.0 | 0.0 | 0.0 | 0.0 | 0.0 | 0.0 | 0.0 |
| LBatSed | 2839.5 | 0.1 | 0.1 | 0.0 | 0.0 | 0.0 | 0.0 | 0.0 | 0.0 | 0.0 | 0.0 | 0.0 | 0.0 |
| Abyss | 247349.7 | 0.0 | 0.0 | 0.0 | 0.0 | 0.0 | 0.0 | 0.0 | 0.0 | 0.0 | 0.0 | 0.0 | 0.0 |
| TOTAL | 312691 | 24510 | 24638 | 7.8 | 7.9 | 100.0 | 100.0 | 5675 | 3090 | 0.32 | 0.2 | 4.3 | 2.3 |

*Appendix S4. verview of Benthic Habitat Types (BHT) in the maritime waters of the Gulf of Cadiz Demarcation. For each BHT, the table presents the total area (km²), trawled area (km²), percentage of BHT area affected by trawling, proportion of the total trawling footprint represented by the BHT, and both total and mean fishing intensity (SAR) supported by the BHT. Fishing activity (trawling footprint) was determined based on average SAR values for the two timeframes, considering SAR values > 0* **y^-1^***. See Supplementary Table 1 for habitat name abbreviations.*

| BHT | AREA (Km^2^) | AREA WITH TRAWLING (Km^2^) | | % HÁBITAT TRAWLED | | % HAB TO TRAW. FOOT | | TOTAL FI (SAR) | | MEAN FI (SAR) TOTAL HABITAT AREA | | MEAN FI (SAR) TRAWLED HABITAT AREA | |
| --- | --- | --- | --- | --- | --- | --- | --- | --- | --- | --- | --- | --- | --- |
|  |  | 2010-2015 | 2016-2022 | 2010-2015 | 2016-2022 | 2010-2015 | 2016-2022 | 2010-2015 | 2016-2022 | 2010-2015 | 2016-2022 | 2010-2015 | 2016-2022 |
| UBatSed | 6708.2 | 2672.0 | 2565.3 | 39.8 | 38.2 | 35.3 | 34.5 | 520.2 | 309.5 | 1.8 | 1.1 | 4.6 | 2.9 |
| CirMu | 1719.4 | 1418.2 | 1418.2 | 82.5 | 82.5 | 18.7 | 19.1 | 836.0 | 749.3 | 11.6 | 10.4 | 14.1 | 12.6 |
| OfCirMu | 1271.9 | 1271.8 | 1271.8 | 100.0 | 100.0 | 16.8 | 17.1 | 766.1 | 599.2 | 14.3 | 11.2 | 14.3 | 11.2 |
| CirSa | 1800.7 | 1067.2 | 1070.0 | 59.3 | 59.4 | 14.1 | 14.4 | 299.3 | 295.6 | 4.0 | 3.9 | 6.7 | 6.6 |
| OfCirSa | 739.8 | 572.4 | 572.4 | 77.4 | 77.4 | 7.6 | 7.7 | 41.8 | 28.7 | 1.3 | 0.9 | 1.7 | 1.2 |
| CirMs | 148.0 | 99.3 | 99.3 | 67.1 | 67.1 | 1.3 | 1.3 | 58.4 | 49.1 | 9.8 | 8.2 | 14.2 | 11.9 |
| UBatRo | 133.5 | 98.9 | 76.3 | 74.1 | 57.1 | 1.3 | 1.0 | 4.2 | 2.0 | 0.8 | 0.4 | 1.1 | 0.6 |
| InfSa | 503.3 | 95.6 | 95.6 | 19.0 | 19.0 | 1.3 | 1.3 | 17.8 | 15.3 | 0.8 | 0.7 | 4.3 | 3.7 |
| CirCs | 100.1 | 85.7 | 85.7 | 85.7 | 85.7 | 1.1 | 1.2 | 10.4 | 7.9 | 2.4 | 1.8 | 2.8 | 2.1 |
| OfCirCs | 83.6 | 79.5 | 79.5 | 95.1 | 95.1 | 1.1 | 1.1 | 7.6 | 6.8 | 2.3 | 2.0 | 2.4 | 2.1 |
| OfCirMs | 56.4 | 56.4 | 56.4 | 100.0 | 100.0 | 0.7 | 0.8 | 44.6 | 36.1 | 20.6 | 16.7 | 20.6 | 16.7 |
| CirRo | 136.4 | 25.0 | 25.0 | 18.3 | 18.3 | 0.3 | 0.3 | 2.0 | 1.5 | 0.4 | 0.3 | 2.0 | 1.5 |
| InfCs | 110.7 | 11.0 | 11.0 | 9.9 | 9.9 | 0.1 | 0.1 | 3.5 | 2.9 | 0.8 | 0.6 | 3.5 | 2.9 |
| InfRo | 153.1 | 9.7 | 9.7 | 6.3 | 6.3 | 0.1 | 0.1 | 0.6 | 0.3 | 0.1 | 0.0 | 0.6 | 0.3 |
| InfMs | 128.4 | 3.7 | 3.7 | 2.9 | 2.9 | 0.0 | 0.1 | 0.0 | 0.0 | 0.0 | 0.0 | 0.0 | 0.0 |
| InfMu | 144.3 | 0.6 | 0.6 | 0.4 | 0.4 | 0.0 | 0.0 | 0.0 | 0.0 | 0.0 | 0.0 | 0.0 | 0.0 |
| OfCirRo | 5.7 | 0.0 | 0.0 | 0.0 | 0.0 | 0.0 | 0.0 | 0.0 | 0.0 | 0.0 | 0.0 | 0.0 | 0.0 |
| UBatS/Ro | 33.1 | 0.0 | 0.0 | 0.0 | 0.0 | 0.0 | 0.0 | 0.0 | 0.0 | 0.0 | 0.0 | 0.0 | 0.0 |
| LBatSed | 0.0 | 0.0 | 0.0 | 0.0 | 0.0 | 0.0 | 0.0 | 0.0 | 0.0 | 0.0 | 0.0 | 0.0 | 0.0 |
| LBatSeR | 20.9 | 0.0 | 0.0 | 0.0 | 0.0 | 0.0 | 0.0 | 0.0 | 0.0 | 0.0 | 0.0 | 0.0 | 0.0 |
| Na | 0.3 | 0.0 | 0.0 | 0.0 | 0.0 | 0.0 | 0.0 | 0.0 | 0.0 | 0.0 | 0.0 | 0.0 | 0.0 |
| TOTAL | 13997.9 | 7566.8 | 7440.3 | 54.1 | 53.2 | 100.0 | 100.0 | 2614.4 | 2105.5 | 4.5 | 3.6 | 8.2 | 6.7 |

Appendix S5. Mean SAR in the study areas during two periods: 2010–2015 (left) and 2016–2022 (right). Top: North Atlantic Demarcation. Bottom: Gulf of Cadiz Demarcation. Yellow-hatched areas indicate regions with no bottom trawling activity, while black-highlighted areas represent zones with bottom trawling activity.

Appendix S6. Pressure-state curves (GAMs) illustrate the relationship between the proportion of sentinel species and bottom trawling intensity (SAR) for each analysed BHT. The solid black line represents the mean relationship, while the grey-shaded area indicates the standard error.

*Appendix S7. Generalised Additive Model (GAM) smooth functions showing the effect of bottom trawling effort on the logit-transformed proportion of sentinel species for each assessed BHT. The solid line represents the estimated smooth, and the shaded area denotes the 95% confidence interval. Grey points indicate the partial residuals, showing the variation of the data around the fitted smooth term.*

*Appendix S8. Prediction error for sentinel species proportions based on mean SAR values for the periods 2010–2015 (left) and 2016–2022 (right) in the study areas. Top: North Atlantic Demarcation. Bottom: Gulf of Cadiz Demarcation. Blue-shaded areas represent BHTs that are unassessed but subject to bottom trawling. Yellow-hatched areas indicate regions with no bottom trawling activity, while black-highlighted areas correspond to zones with bottom trawling activity (trawling footprint, SAR > 0 y^-1^).*

*Appendix S9***. Sensitivity of the BHT analysed calculated based on Plaza-Morlote, 2023 and Serrano et al., 2022.**

| **BHT** | **SENSITIVITY VALUE** |
| --- | --- |
| **NORTH ATLANTIC DEMARCATION** | |
| UBatSed | 3.71 ± 0.45 |
| OfCirSa | 3.27 ± 0.45 |
| OfCirMu | 2.95 ± 0.64 |
| OfCirMs | 3.08 ± 0.28 |
| **GULF OF CADIZ DEMARCATION** | |
| UBatSed | 4.00 ± 0.00 |

*Appendix S10.. Mean proportion of sentinel species (mean benthic status) per BHT in total and trawled areas.^*^*

| BHT | MEAN BENTHIC STATUS TOTAL HABITAT AREA | | MEAN BENTHIC STATUS TRAWLED AREA | |
| --- | --- | --- | --- | --- |
|  | 2010-2015 | 2016-2022 | 2010-2015 | 2016-2022 |
| NORTH ATLANTIC DEMARCATION | | | | |
| All BHTs assessed | 0.53 | 0.67 | 0.40 | 0.58 |
| UBatSed | 0.63 | 0.72 | 0.41 | 0.56 |
| OfCirSa | 0.53 | 0.69 | 0.51 | 0.67 |
| OfCirMu | 0.23 | 0.49 | 0.22 | 0.48 |
| OfCirMs | 0.39 | 0.60 | 0.35 | 0.57 |
| GULF OF CADIZ DEMARCATION | | | | |
| UBatSed | 0.74 | 0.80 | 0.35 | 0.49 |

^*^ The status is presented for the total area of each BHT as well as for the trawled portions, which vary in extent between habitats (see Supplementary Tables 2 and 3). For habitat name abbreviations, refer to Supplementary Table 1.

*Appendix S11. Comparison of state and pressure thresholds across three evaluation approaches^*^.*

| **BHT** | **DISTANCE TO DEGRADATION** | | **NATURAL VARIATION** | | **DETECTABLE CHANGE** | |
| --- | --- | --- | --- | --- | --- | --- |
|  | PRESSURE THRESHOLD | STATE  THRESHOLD/ | PRESSURE THRESHOLD | STATE  THRESHOLD | PRESSURE THRESHOLD | STATE  THRESHOLD |
| **NORTH ATLANTIC DEMARCATION** | | | | | |  |
| UBatSed | 0.89 | 0.81 | 1.14 | 0.76 | 1.86 | 0.63 |
| OfCirSa | 2.18 | 0.63 | 1.31 | 0.77 | 2.12 | 0.64 |
| OfCirMu | 1.37 | 0.67 | 1.97 | 0.55 | 2.75 | 0.43 |
| OfCirMs | 1.99 | 0.64 | 1.18 | 0.88 | 2.05 | 0.76 |
| **GULF OF CADIZ DEMARCATION** | | | | | |  |
| UBatSed | 0.46 | 0.78 | 0.88 | 0.61 | 1.51 | 0.42 |

*^*^ Quality thresholds were derived using the Distance to Degradation, Natural Variability, and Detectable Change methods. State thresholds correspond to the SoS indicator; pressure thresholds refer to trawling intensity (SAR).*

*Appendix S12. Derivation of quality thresholds using three different approaches. Solid green, blue and orange lines represent the thresholds distinguishing "good" from "degraded" quality, as well as pressure and state quality thresholds. Grey lines and points denote additional reference values used in threshold derivation. For the degradation point approach, the model is shown with standard error, whereas in the natural variation and detectable change approaches, the correlation model is displayed with 95% confidence intervals*

Appendix S13. Summary of the extent of adverse effects (impacts) to trawling assessment statistics.

| **BHT** | **TOTAL AREA (Km^2^)** | **THRESHOLD** | **PERIOD** | **ASSESSMENT STATUS** | **AREA (Km^2^)** | **PROPORTION (%)** |
| --- | --- | --- | --- | --- | --- | --- |
| **NORTH ATLANTIC DEMARCATION** | | | | | | |
| ALL ANALYSED HABITATS  IN NIA | 25541.8 | DEGRADATION POINT | 2010-2015 | NO PRESSURE | 5476.89 | 21.44 |
|  |  |  |  | NOT ADVERSELY AFFECTED | 4602.83 | 18.02 |
|  |  |  |  | ADVERSELY AFFECTED | 15462.07 | 60.54 |
|  |  |  | 2016-2022 | NO PRESSURE | 5373.83 | 21.04 |
|  |  |  |  | NOT ADVERSELY AFFECTED | 7450.41 | 29.17 |
|  |  |  |  | ADVERSELY AFFECTED | 12717.56 | 49.79 |
|  |  | NATURAL VARIATION | 2010-2015 | NO PRESSURE | 5476.89 | 21.44 |
|  |  |  |  | NOT ADVERSELY AFFECTED | 4126.54 | 16.16 |
|  |  |  |  | ADVERSELY AFFECTED | 15938.37 | 62.40 |
|  |  |  | 2016-2022 | NO PRESSURE | 5373.83 | 21.04 |
|  |  |  |  | NOT ADVERSELY AFFECTED | 6930.98 | 27.14 |
|  |  |  |  | ADVERSELY AFFECTED | 13236.99 | 51.82 |
|  |  | DETECTABLE CHANGE | 2010-2015 | NO PRESSURE | 5476.89 | 21.44 |
|  |  |  |  | NOT ADVERSELY AFFECTED | 6191.68 | 24.24 |
|  |  |  |  | ADVERSELY AFFECTED | 13873.23 | 54.32 |
|  |  |  | 2016-2022 | NO PRESSURE | 5373.83 | 21.04 |
|  |  |  |  | NOT ADVERSELY AFFECTED | 10038.05 | 39.30 |
|  |  |  |  | ADVERSELY AFFECTED | 10129.92 | 39.66 |
| UBatSed | 13127.7 | DEGRADATION POINT | 2010-2015 | NO PRESSURE | 4949.14 | 37.70 |
|  |  |  |  | NOT ADVERSELY AFFECTED | 2117.83 | 16.13 |
|  |  |  |  | ADVERSELY AFFECTED | 6060.73 | 46.17 |
|  |  |  | 2016-2022 | NO PRESSURE | 4830.99 | 36.80 |
|  |  |  |  | NOT ADVERSELY AFFECTED | 2570.57 | 19.58 |
|  |  |  |  | ADVERSELY AFFECTED | 5726.14 | 43.62 |
|  |  | NATURAL VARIATION | 2010-2015 | NO PRESSURE | 4949.14 | 37.70 |
|  |  |  |  | ADVERSELY AFFECTED | 2266.84 | 17.27 |
|  |  |  |  | NOT ADVERSELY AFFECTED | 5911.72 | 45.03 |
|  |  |  | 2016-2022 | NO PRESSURE | 4830.99 | 36.80 |
|  |  |  |  | ADVERSELY AFFECTED | 2853.85 | 21.74 |
|  |  |  |  | NOT ADVERSELY AFFECTED | 5442.86 | 41.46 |
|  |  | DETECTABLE CHANGE | 2010-2015 | NO PRESSURE | 4949.14 | 37.70 |
|  |  |  |  | NOT ADVERSELY AFFECTED | 2635.65 | 20.08 |
|  |  |  |  | ADVERSELY AFFECTED | 5542.91 | 42.22 |
|  |  |  | 2016-2022 | NO PRESSURE | 4830.99 | 36.80 |
|  |  |  |  | NOT ADVERSELY AFFECTED | 3626.68 | 27.63 |
|  |  |  |  | ADVERSELY AFFECTED | 4670.03 | 35.57 |
| OfCirSa | 7545.2 | DEGRADATION POINT | 2010-2015 | NO PRESSURE | 399.90 | 5.30 |
|  |  |  |  | NOT ADVERSELY AFFECTED | 2415.41 | 32.01 |
|  |  |  |  | ADVERSELY AFFECTED | 4729.89 | 62.69 |
|  |  |  | 2016-2022 | NO PRESSURE | 414.99 | 5.50 |
|  |  |  |  | NOT ADVERSELY AFFECTED | 4115.07 | 54.54 |
|  |  |  |  | ADVERSELY AFFECTED | 3015.14 | 39.96 |
|  |  | NATURAL VARIATION | 2010-2015 | NO PRESSURE | 399.90 | 5.30 |
|  |  |  |  | NOT ADVERSELY AFFECTED | 1742.31 | 23.09 |
|  |  |  |  | ADVERSELY AFFECTED | 5402.99 | 71.61 |
|  |  |  | 2016-2022 | NO PRESSURE | 414.99 | 5.50 |
|  |  |  |  | NOT ADVERSELY AFFECTED | 2873.19 | 38.08 |
|  |  |  |  | ADVERSELY AFFECTED | 4257.03 | 56.42 |
|  |  | DETECTABLE CHANGE | 2010-2015 | NO PRESSURE | 399.90 | 5.30 |
|  |  |  |  | NOT ADVERSELY AFFECTED | 2378.23 | 31.52 |
|  |  |  |  | ADVERSELY AFFECTED | 4767.08 | 63.18 |
|  |  |  | 2016-2022 | NO PRESSURE | 414.99 | 5.50 |
|  |  |  |  | NOT ADVERSELY AFFECTED | 3955.66 | 52.43 |
|  |  |  |  | ADVERSELY AFFECTED | 3174.55 | 42.07 |
| OfCirMu | 4220.5 | DEGRADATION POINT | 2010-2015 | NO PRESSURE | 84.41 | 2.00 |
|  |  |  |  | NOT ADVERSELY AFFECTED | 0.00 | 0.00 |
|  |  |  |  | ADVERSELY AFFECTED | 4136.09 | 98.00 |
|  |  |  | 2016-2022 | NO PRESSURE | 84.41 | 2.00 |
|  |  |  |  | NOT ADVERSELY AFFECTED | 536.69 | 12.72 |
|  |  |  |  | ADVERSELY AFFECTED | 3599.40 | 85.28 |
|  |  | NATURAL VARIATION | 2010-2015 | NO PRESSURE | 84.41 | 2.00 |
|  |  |  |  | NOT ADVERSELY AFFECTED | 85.93 | 2.04 |
|  |  |  |  | ADVERSELY AFFECTED | 4050.16 | 95.96 |
|  |  |  | 2016-2022 | NO PRESSURE | 84.41 | 2.00 |
|  |  |  |  | NOT ADVERSELY AFFECTED | 1086.93 | 25.75 |
|  |  |  |  | ADVERSELY AFFECTED | 3049.16 | 72.25 |
|  |  | DETECTABLE CHANGE | 2010-2015 | NO PRESSURE | 84.41 | 2.00 |
|  |  |  |  | NOT ADVERSELY AFFECTED | 1108.21 | 26.26 |
|  |  |  |  | ADVERSELY AFFECTED | 3027.88 | 71.74 |
|  |  |  | 2016-2022 | NO PRESSURE | 84.41 | 2.00 |
|  |  |  |  | NOT ADVERSELY AFFECTED | 2224.23 | 52.70 |
|  |  |  |  | ADVERSELY AFFECTED | 1911.86 | 45.30 |
| OfCirMs | 648.4 | DEGRADATION POINT | 2010-2015 | NO PRESSURE | 43.44 | 6.70 |
|  |  |  |  | NOT ADVERSELY AFFECTED | 69.59 | 10.73 |
|  |  |  |  | ADVERSELY AFFECTED | 535.36 | 82.57 |
|  |  |  | 2016-2022 | NO PRESSURE | 43.44 | 6.70 |
|  |  |  |  | NOT ADVERSELY AFFECTED | 228.08 | 35.18 |
|  |  |  |  | ADVERSELY AFFECTED | 376.88 | 58.12 |
|  |  | NATURAL VARIATION | 2010-2015 | NO PRESSURE | 43.44 | 6.70 |
|  |  |  |  | NOT ADVERSELY AFFECTED | 31.46 | 4.85 |
|  |  |  |  | ADVERSELY AFFECTED | 573.50 | 88.45 |
|  |  |  | 2016-2022 | NO PRESSURE | 43.44 | 6.70 |
|  |  |  |  | NOT ADVERSELY AFFECTED | 117.01 | 18.05 |
|  |  |  |  | ADVERSELY AFFECTED | 487.94 | 75.25 |
|  |  | DETECTABLE CHANGE | 2010-2015 | NO PRESSURE | 43.44 | 6.70 |
|  |  |  |  | NOT ADVERSELY AFFECTED | 69.59 | 10.73 |
|  |  |  |  | ADVERSELY AFFECTED | 535.36 | 82.57 |
|  |  |  | 2016-2022 | NO PRESSURE | 43.44 | 6.70 |
|  |  |  |  | NOT ADVERSELY AFFECTED | 231.48 | 35.70 |
|  |  |  |  | ADVERSELY AFFECTED | 373.48 | 57.60 |
|  | | | | | | |
| UBatSed | 6708.2 | DEGRADATION POINT | 2010-2015 | NO PRESSURE | 4038.34 | 60.20 |
|  |  |  |  | NOT ADVERSELY AFFECTED | 466.71 | 6.96 |
|  |  |  |  | ADVERSELY AFFECTED | 2203.16 | 32.84 |
|  |  |  | 2016-2022 | NO PRESSURE | 4145.67 | 61.80 |
|  |  |  |  | NOT ADVERSELY AFFECTED | 658.02 | 9.81 |
|  |  |  |  | ADVERSELY AFFECTED | 1904.51 | 28.39 |
|  |  | NATURAL VARIATION | 2010-2015 | NO PRESSURE | 4038.34 | 60.20 |
|  |  |  |  | NOT ADVERSELY AFFECTED | 671.31 | 10.01 |
|  |  |  |  | ADVERSELY AFFECTED | 1998.55 | 29.79 |
|  |  |  | 2016-2022 | NO PRESSURE | 4145.67 | 61.80 |
|  |  |  |  | NOT ADVERSELY AFFECTED | 941.56 | 14.04 |
|  |  |  |  | ADVERSELY AFFECTED | 1620.98 | 24.16 |
|  |  | DETECTABLE CHANGE | 2010-2015 | NO PRESSURE | 4038.34 | 60.20 |
|  |  |  |  | NOT ADVERSELY AFFECTED | 950.13 | 14.16 |
|  |  |  |  | ADVERSELY AFFECTED | 1719.73 | 25.64 |
|  |  |  | 2016-2022 | NO PRESSURE | 4145.67 | 61.80 |
|  |  |  |  | NOT ADVERSELY AFFECTED | 1445.10 | 21.54 |
|  |  |  |  | ADVERSELY AFFECTED | 1117.43 | 16.66 |

Appendix S14. Cross-validation metrics for GAMs predicting the SoS indicator across BHTs and regions.

| BHT | SIGNIFICANT ITERATIONS (%) | MEAN RHO | SD RHO | |
| --- | --- | --- | --- | --- |
| NORTH ATLANTIC DEMARCATION | | | |  |
| UBatSed | 100 | 0.57 | 0.07 | |
| OfCirSa | 80 | 0.36 | 0.16 | |
| OfCirMu | 70 | 0.25 | 0.14 | |
| OfCirMs | 100 | 0.66 | 0.13 | |
| GULF OF CADIZ DEMARCATION | | | | |
| UBatSed | 100 | 0.44 | 0.09 | |

^*^ The percentage of significant iterations (%) indicates the proportion of cross-validation repetitions (out of 10) in which Spearman’s rank correlation (rho) between observed and predicted SoS values was statistically significant (p < 0.05). Mean rho and standard deviation (SD) rho summarise the average strength and variability of the correlation across all iterations.
